# Optic Flow Stimuli in and Near the Visual Field Centre: A Group fMRI Study of Motion Sensitive Regions

**DOI:** 10.1371/journal.pone.0004043

**Published:** 2008-12-29

**Authors:** Sabine Ohlendorf, Andreas Sprenger, Oliver Speck, Sven Haller, Hubert Kimmig

**Affiliations:** 1 Neurologische Klinik, Universitätsklinikum, Freiburg, Germany; 2 Klinik für Neurologie, Universitätsklinikum Schleswig Holstein, Campus Lübeck, Lübeck, Germany; 3 Abteilung Röntgendiagnostik, Medizin Physik, Universitätsklinikum, Freiburg, Germany; 4 Abteilung Biomedizinische Magnetresonanz, Institut für Experimentelle Physik, Universität Magdeburg, Magdeburg, Germany; 5 Abteilung für Neuroradiologie, Universitätsspital, Basel, Switzerland; 6 Fakultät für Biologie, Universität Freiburg, Freiburg, Germany; 7 Schwarzwald-Baar-Klinikum, Academic Teaching Hospital Universität Freiburg, Villingen-Schwenningen, Germany; University of Chicago, United States of America

## Abstract

Motion stimuli in one visual hemifield activate human primary visual areas of the contralateral side, but suppress activity of the corresponding ipsilateral regions. While hemifield motion is rare in everyday life, motion in both hemifields occurs regularly whenever we move. Consequently, during motion primary visual regions should simultaneously receive excitatory and inhibitory inputs. A comparison of primary and higher visual cortex activations induced by bilateral and unilateral motion stimuli is missing up to now. Many motion studies focused on the MT+ complex in the parieto-occipito-temporal cortex. In single human subjects MT+ has been subdivided in area MT, which was activated by motion stimuli in the contralateral visual field, and area MST, which responded to motion in both the contra- and ipsilateral field. In this study we investigated the cortical activation when excitatory and inhibitory inputs interfere with each other in primary visual regions and we present for the first time group results of the MT+ subregions, allowing for comparisons with the group results of other motion processing studies. Using functional magnetic resonance imaging (fMRI), we investigated whole brain activations in a large group of healthy humans by applying optic flow stimuli in and near the visual field centre and performed a second level analysis. Primary visual areas were activated exclusively by motion in the contralateral field but to our surprise not by central flow fields. Inhibitory inputs to primary visual regions appear to cancel simultaneously occurring excitatory inputs during central flow field stimulation. Within MT+ we identified two subregions. Putative area MST (pMST) was activated by ipsi- and contralateral stimulation and located in the anterior part of MT+. The second subregion was located in the more posterior part of MT+ (putative area MT, pMT).

## Introduction

In human imaging studies, the most significant activations related to motion processing have been found in the lingual region (ventral V3), in V3A, in the lateral occipital region (LOS), in the MT+ complex and in the intraparietal sulcus (IPS) of the posterior parietal cortex [cf. 1]. All these regions get direct or indirect input from the primary visual area (V1) [2], [3]. In some fMRI studies investigating cortical processing of optic flow, V1 was activated [4], in others it was not activated [1] but its role in motion processing has never been discussed in that context. Visual motion (compared to stationary visual stimulation) in one hemifield seems to activate primary visual cortex of the contralateral side, but to suppress activation of the corresponding ipsilateral regions [5], [6]. In everyday life, flow field motion occurs regularly in both visual hemifields, whenever we move. Therefore, during motion primary visual regions should receive both excitatory and inhibitory inputs, and fMRI activations in primary visual regions would be expected to be the result of such competing inputs. A comparison of bilateral motion stimuli (in both hemifields) with unilateral motion stimuli (in one hemifield) is however missing up to now.

A well-known motion processing region, MT+ has been identified in humans by positron emission tomography [7], [8], histological methods [9], [10] and fMRI studies [11]–[14]. Human MT+ is located on the ascending limb of the inferior temporal sulcus. Until to date few fMRI studies investigated the human MT+ subdivision in MT and MST, their locations, size and functional properties. Subarea MT has been shown to be located in a more posterior part and subarea MST in a more anterior part of the MT+ complex. Morrone et al. [15] identified a ventral subarea of the MT+ complex which was especially sensitive to direction changing optic flow. Dukelow et al. [16] and Huk et al. [17] identified subarea MST which was activated by ipsilateral and contralateral flow field stimulation. MT was exclusively activated by contralateral stimulation and defined as the non-MST part of MT+. Furthermore, MT but not MST seemed to respond to retinotopic stimulation [17]. A subarea of MST was presumed to transform optic flow into head centric flow [4]; also MT has been shown to get spatial input [18]. To explain the effect that MST was activated by contralateral as well as ipsilateral stimulation while MT responded exclusively to contralateral stimulation, it is usually referred to the different receptive field sizes of monkey MT and MST neurons. In monkey, MT neurons were shown to have small receptive fields (RF). RF of MT neurons close to the midline expanded about 10–15° into the ipsilateral visual field [19], [20], while MSTd neurons with large RF covered most of the ipsilateral field [21]. All cited human fMRI studies measured peripheral large field motion stimuli during central fixation without measuring eye movements in the scanner. Furthermore, most of the human fMRI studies investigated few subjects (n = 4–9) with considerable intersubject variability, so that the data analysis was focused on the presentation of single subject maps of MT+. An inference on the population level was not possible.

In this study we measured whole brain fMRI activations in a large group of healthy humans. Optic flow stimuli were applied in the visual field centre (bilateral stimulation) and near the field centre (unilateral stimulation). Thus we were able to measure the outcome of competing excitatory and inhibitory inputs to primary visual regions and to present for the first time group results of the MT+ subregions, allowing for comparisons with the group results of other motion processing studies.

## Materials and Methods

### Visual Stimulation

Visual stimuli were programmed using Matlab (The Mathworks, Natick, USA) in combination with Cogent Graphics (developed by J. Romaya, at the LON at the Wellcome Department of Imaging Neuroscience, UK) and back-projected onto a transluminent screen via an LCD-projector (NEC MT 1050, Tokyo, Japan; 1024×768 spatial resolution at 60 Hz). The screen was placed in the gantry at a distance of 75 cm to the subjects' eyes (30°×23° of visual angle). Subjects saw the visual stimulation via a mirror which was mounted on the MR-headcoil. Great care was taken to completely darken the room and to avoid stray light. The light of the projector was substantially reduced by two polarizing filters and by darkening the translucent screen thereby reducing the stimulus contrast. The stimulation paradigm was based on the one described by Huk et al. [17]. Stimuli consisted in alternating moving and stationary dot patterns of circular shape. In contrast to their stimuli we used slow optic flow, and unilateral stimulation was located near to the central fixation location.

### Bilateral flow stimulus

**Figure 1:**
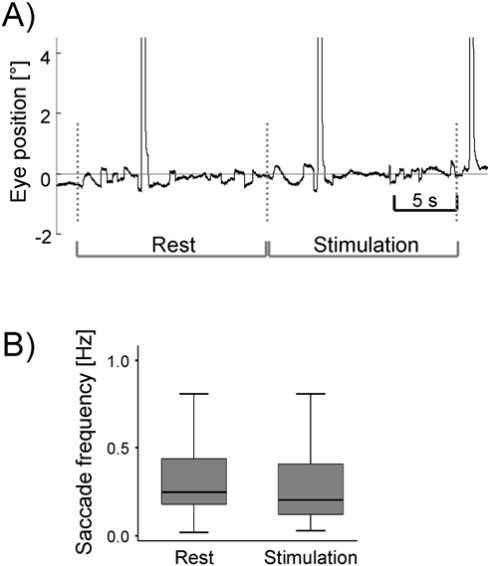
Eye movement data. (A) Original eye position trace of one subject in a rest and consecutive stimulation period. Leftward eye movements are shown as negative values. Small saccades as well as blinks (large positive excursions) are present in both rest and stimulation periods. (B) Saccadic frequency of n = 18 subjects in the rest and stimulation conditions.

A red fixation dot was projected in the centre of the screen (Ø 0.3° of visual angle). Subjects had to fixate this central dot during the whole scanning session (7.5 min). White dots (n = 150; Ø 0.3°) were randomly distributed in a circular area (radius 8°). In the rest condition, the white dots remained stationary (duration 15 s). In the stimulation condition (duration 15 s), the dots moved radially from appearance at the centre of the flow field to disappearance at the pattern periphery with increasing speed of 0.5 to 4.2°/s (mean velocity 2.5°/s), and changed direction once per second. The central location of the flow pattern stimulated motion sensitive regions bilaterally, i.e. in both hemifields. The dot pattern was spared around the fixation dot (min. radius 1.2°; Fig. 1A).

### Unilateral flow stimulus

The circular dot pattern was located either in the right or left visual hemifield (offset of centre of the circular pattern from the mid-line 8°; max. radius of the dot pattern 6°; min. radius 1.2°, shortest distance from the dot patch to the fixation dot, 2°; amount of white dots = 113; Fig. 1B, C). In the stimulation condition, we used the same velocity profiles as in the bilateral flow task.

Each flow stimulus was compared with its own corresponding rest condition consisting of stationary dots (i.e. the right hemifield optic flow stimulus was compared with the right hemisphere rest stimulus, the left hemifield optic flow stimulus was compared with the left hemisphere rest stimulus and the bilateral optic flow stimulus was compared with the bilateral rest stimulus). These pairs of stimuli occurred in a pseudo randomized order. Each stimulation type and its rest condition (Bilateral flow stimulus, unilateral flow stimulus +8°, unilateral flow stimulus −8°) were repeated five times in the fMRI session (duration 7.5 min).

### MR Eyetracking

For horizontal eye movement recordings we used the Freiburg MR-Eyetracker system, a fiber-optic limbus tracking device [22]. A multi-channel computer program (LabVIEW®, National Instruments, Austin, USA) recorded the eye movement data. The sampling frequency was 500 Hz, the spatial resolution 0.2° of visual angle. Deviation from linearity for ±20° was less than 5%. For calibration, subjects shifted their eyes repeatedly from the central fixation point towards targets at lateral locations of ±5°.

### MR-Imaging

Magnetic resonance imaging was performed with a 3 Tesla Magnetom TRIO scanner (Siemens, Erlangen, Germany). Functional imaging was performed with a T2*-weighted echo-planar imaging (EPI) sequence which was equipped with fully automated distortion correction [23]. High-resolution, sagittal T1-weighted images were acquired with the MP-RAGE (magnetization prepared rapid acquisition gradient echo) sequence to obtain a 3D anatomical scan of the brain. The technical data for the functional measurements were TE 30 ms, TR 2.5 s, flip angle 90°, field of view 192 * 210 mm^2^, matrix 64×70, voxel size 3*3*3 mm^3^. The stimulation protocol consisted of thirty 15 s intervals including 15 periods of rest (OFF) and 15 periods of stimulation (ON). This protocol produced 180 echo planar volumes in one series (duration 7.5 min). Data acquisition was performed in 36 slices per volume containing the whole brain excluding the cerebellum. To minimize head motion, the subject's head was fixed in the MR headcoil. Gradient noises were reduced by sound-dampening headphones.

### Subjects

Eighteen healthy subjects (17 right handed and one left handed, age range 18–35 years) were included in the data analysis. Subjects' vision was normal or corrected to normal. Written informed consent was obtained from all subjects. The study was approved by the local Ethics Committee of the University of Freiburg.

### Eye movement data analysis

Since visual stimulation in this experiment did not include any tasks of eye movements but required exclusively fixation of a stationary dot, the eye movement data were used to control for the subjects' vigilance and permanent fixation of the central dot.

### fMRI data analysis

FMRI data were analyzed by use of the software package SPM5 (Wellcome Department of Cognitive Neurology, London, UK). Residual head motion was corrected via SPM5 realignment. For multiple comparisons we normalized the EPI volumes using white and gray matter segmentation parameters of the anatomical T1 image. Spatial smoothing was performed with Gaussian spatial kernels of 8 mm (full width at half maximum). For statistical analysis data were fitted to a general linear model to establish parameter estimates for each subject.

We defined the main contrast for the bilateral flow condition. For the unilateral flow conditions we defined main contrasts for stimulation of both the left and the right visual hemifield (all conditions were calculated moving dots – stationary dots) on a single subject level. For group comparisons we included the resulting main contrast images into three random effects one sample t-tests and corrected for multiple comparisons using family wise error (FWE) correction. Clusters of adjacent voxels surpassing an individual threshold of p = 0.05 (corrected) were considered as significant activations.

For visualization purposes we projected the functional group results onto the left and right hemispheres of the human Colin surface-based atlas mapped to PALS (‘Population-Average Landmark- and Surface-based’-atlas; [24]–[26]. Data were mapped on the flatmap template and the three dimensional cortical template of the atlas. This was done using the Computerized Anatomical Reconstruction and Editing Toolkit (CARET) version 5.3 (http://brainvis.wustl.edu [27]). Statistical representations of the three main effects were mapped to different colors in functional overlays. Co-activated regions were displayed by weighted additive color while pure colors indicated regions activated by only one of the tasks (Bilateral flow stimulus – blue; unilateral flow stimulus, ipsilateral – red, contralateral – green; intensity scale 0–255 referring to the maximum activation of each contrast). Note that the flatmaps were only used for purposes of visualization of activation locations. They exclusively show grey matter activations surpassing a minimum threshold of T = 6 without considering effect size differences of stimulation tasks.

We report all findings in the MNI (Montreal Neurological Institute) coordinate system. Anatomic activation localization was performed via the SPM5 tool ‘wfu pickatlas’ [28], [29], functional localizations were performed in agreement with the anatomy toolbox of SPM [30] and geared to functional borders of the Human PALS atlas [26] which both are based on group results of previous publications. Interindividual differences of our data were taken into account by the SPM second level group analysis. Cerebellar and brainstem activations were not included in the statistical analysis.

## Results

### Eye movement data

During the whole experiment subjects fixated the centre dot correctly and did not make saccades to the stimuli shown in the left or right visual hemifield. Small corrective saccades (amplitude less than 1°) were equally distributed across motion and rest conditions (mean frequency±standard deviation in the rest conditions 0.37 Hz±0.33, 0.31 Hz±0.27 in motion conditions, no significant difference p = 0.5; Fig. 1AB). Since we calculated all our fMRI contrasts as activation contrasts versus the rest condition, we assume that saccade-related cortical activity plays a negligible role in all our contrasts.

### fMRI data

**Figure 2:**
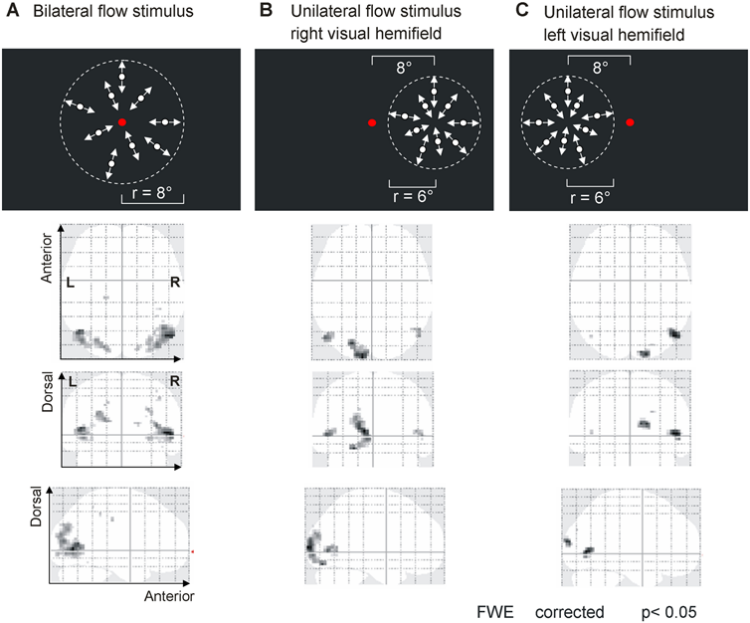
Schematic drawing of stimuli and corresponding cortical activations. First row: (A) Bilateral flow stimulus, (B) Unilateral flow stimulus in the right visual hemifield, (C) Unilateral flow stimulus in the left visual hemifield. 2–4^th^ row (glassbrains; cortical activation contrasts calculated vs. corresponding rest conditions) (A): bilateral flow stimulus leads to activation in MT+ and additional occipito-parietal activation in both hemispheres. (B): unilateral flow in right visual hemifield leads to stronger activation in the left MT+ area, weaker activation in the right MT+ area (pMST) and additional activation in primary visual areas of the left hemisphere. (C): unilateral flow in the left visual hemifield leads to stronger activation in the right MT+ area, very weak activation in the left MT+ area (pMST) and activation in primary visual areas of the right hemisphere. Shown activations are FWE corrected, n = 18. Glassbrain presentation of data in axial coronal and sagittal planes (for maximum t-values compare table 1).

The bilateral flow stimulus led to bilateral activations in the medial occipital gyrus and middle temporal gyrus, corresponding to the MT+ region on group level (random effects group analysis, one sample t-test; FWE corrected; table 1; Fig. 2A). Furthermore, we found activations in parts of V3A, V7, the lateral occipital sulcus (LOS) and a very small activation in the intraparietal sulcus (inferior parietal lobule).

**Table 1:** Activation locations of main contrasts (stimulation (Stim.) vs. rest).

Anatomical Area	Localizer Stim. vs. Rest						Ipsilateral Stim. in Right Visual Hemifield						Ipsilateral Stim. in Left Visual Hemifield					
	BA/fR	Coordinates			CS	T	vs. Rest in Right Visual Hemifield						vs. Rest in Left Visual Hemifield					
		X	Y	Z			BA/fR	Coordinates			CS	T	BA/fR	Coordinates			CS	T
								X	Y	Z				X	Y	Z		
R Mid. Temp. Gyr.	BA 37 (MT+)	51	−69	3	222	13.5	BA 37 (MT+)	54	−69	0	25	8.7	BA 37 (MT+)	48	−69	3	56	11.4
		45	−60	3	222	10.3	BA39 (MT+)	48	−60	6	25	8.2						
L Mid. Temp. Gyr.	BA 39 (MT+)	−45	−63	9	94	11.8							BA 37 (MT+)	−42	−69	3	3	7.6
R Mid. Occ. Gyr.		39	−81	12	222	10.2												
	BA 18 (V3A)	30	−87	−3	15	8.4												
L Mid. Occ. Gyr.	BA 19 (MT+)	−42	−75	8	94	9.8	BA 19 (MT+)	−51	−72	3	34	9.5						
	BA 19 (V3A)	−33	−81	12	94	7.3												
		−27	−75	27	56	9.5												
R Cuneus	(V3A)	18	−87	15	5	8.2							BA 18 (V1/V2)	18	−93	15	40	10.4
L Cuneus	(V3A)	−18	−84	21	56	9.5	BA 18 (V1/V2)	−9	−96	6	196	11.4	BA7	24	−81	30	2	7.7
R Precuneus	BA 31	30	−81	33	18	8.7												
L Precuneus	BA 19 (V8/LOS)	−33	−75	−6	7	7.6												
R Inf. Par. Lob.	BA 40	42	−36	48	3	8												
L Lingual Gyrus							BA 18 (V3v)	−21	−78	−15	196	10.3						
							BA 17 (V1/V2)	−6	−90	−3	196	9.2						

Coordinates show the local maximum of an activated voxel cluster in MNI space; BA = Brodmann Area; fR = supposed functional region (V1/V2 means located in V1 or V2); T = T-value of maximum activated voxel, CS = cluster size, Mid. Temp. Gyr. = Middle Temporal Gyrus, Mid. Occ. Gyr. = Middle Occipital Gyrus, Inf. Par. Lob = Inferior Parietal Lobule.

The group analysis of the unilateral flow stimulus in the right visual hemifield showed activation in the left/contralateral MT+ region and a smaller activation in the right/ipsilateral MT+ region. Furthermore, we found activation in the left/contralateral cuneus and the left lingual gyrus corresponding to primary visual cortex V1/V2 (table 1; Fig. 2B).

The group analysis of the unilateral flow stimulus in the left visual hemifield showed activation in the right/contralateral MT+ region and only very small activation in the left/ipsilateral MT+ region. Further significant activations were located in the right cuneus corresponding to primary visual cortex V1/V2 (table 1; Fig. 2C).

**Figure 3:**
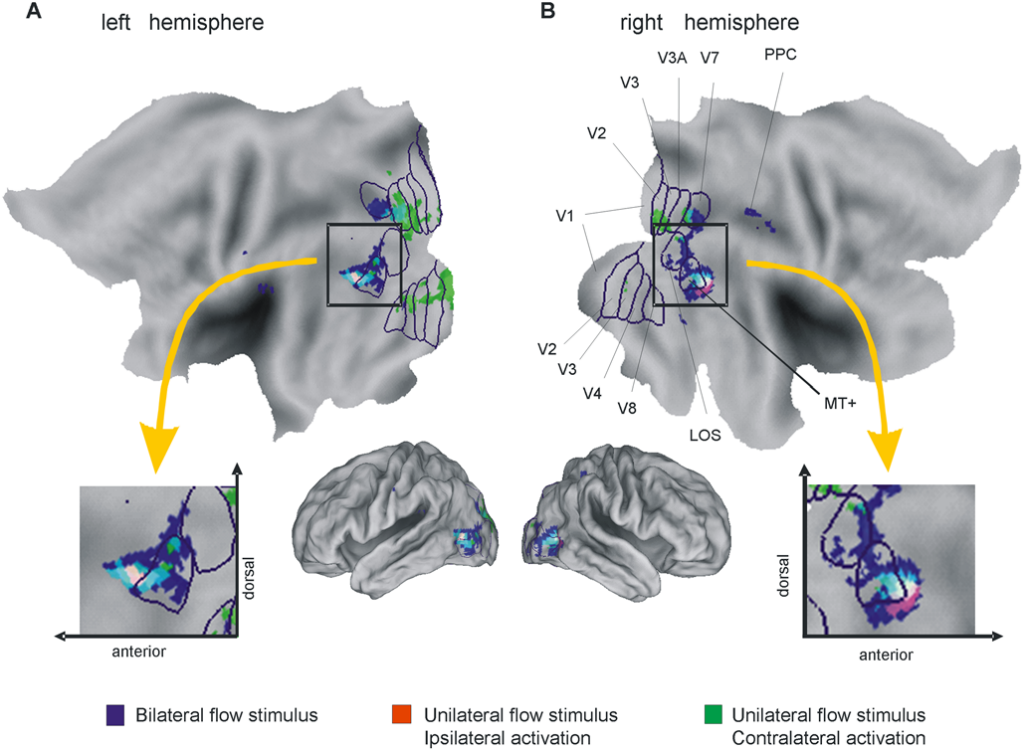
Flatmaps of (A) the left and (B) the right hemisphere of the human PALS Atlas. Functional data overlaid on the flattened template brain. Functional data are RGB coded, intensity scaled to arbitrary values between 0–255. Blue, bilateral flow stimulus activations; red, unilateral flow stimulus - ipsilateral activations; green, unilateral flow stimulus - contralateral activations. Mixed colors show overlay of activations. T-threshold = 6. Insets show enlarged sections of the MT+ complex. For ease of interpretation known human visual areas are outlined in blue, taken from human PALS atlas [26], and a lateral view on the slightly inflated 3D PALS template is given.

The local distribution of task-dependent activations was visualized by calculating flat maps with overlays of all three contrast types (bilateral flow stimulus – blue; unilateral stimulus/ipsilateral activation – red; unilateral stimulus/contralateral activation – green). The above stated activations in MT+ were all located within the activated areas of the bilateral flow stimulus (Fig. 3AB, depicted in blue). In the right hemisphere, they were clearly separated in a more anterior, ipsilateral activation (Fig. 3B red) and an adjacent, more posterior, contralateral activation in the MT+ region (Fig. 3B, green on blue). In the centre of activation the two subareas had a small area of overlap (Fig. 3B, whitish). In the left hemisphere ipsilateral activation was similarly located in the frontal part of the MT+ region (Fig. 3A, red – whitish), however it was not separated from the contralateral activation which spread out further to the anterior part of MT+ (Fig. 3A).

The bilateral flow stimulus did not lead to any activation in visual areas lower than V3A, especially the primary visual cortex. In contrast, the unilateral flow stimulus led to contralateral activation of V1. Furthermore, during stimulation in the ipsilateral visual hemifield we did not find any activation outside MT+.

## Discussion

In this study we investigated motion sensitive cortical regions using optic flow stimuli located in the centre of vision (bilateral flow stimulus) and in a circumscribed area of the right and left visual hemifield close to the midline (unilateral stimuli). Remind that all motion stimuli were compared to the corresponding stationary stimuli, right visual hemifield optic flow to right static visual hemifield, left visual hemifield optic flow to left static visual hemifield, bilateral optic flow to bilateral static visual field. Subjects performed central fixation correctly during the whole stimulation period. Small corrective saccades were equally distributed across motion and rest conditions such that saccade related activations became irrelevant in all our contrast calculations.

Our unilateral motion stimulus activated the contralateral visual areas V1, V2 in the left hemisphere and area V2 in the right hemisphere [in line with 5], [6], [21] and MT+ bilaterally. Side differences of activations in V1 and V2 could be due to threshold effects (in one case activations could remain sub-threshold and do not get significant in the other case they could be just above the threshold and become significant), we suggest not to overestimate these side-differences in our data set. Our bilateral flow stimulus activated parts of V3A, V7, LOS, MT+ and the intraparietal sulcus. Surprisingly, we did not find any activation below V3A, especially V1 was not significantly activated in line with [1]. Goosens et al. [4] in contrast reported V1 activation resulting from a large optic flow stimulus. One explanation might be the contrast of our stimuli (see methods). Previously, Tootell et al. [31] showed that low contrast stimuli activated only MT+ and V3A, in line with our data. Furthermore, Tootell and Taylor [10] showed that MT+ and V3A are more sensitive to motion than lower visual areas. In the study of Maruyama et al. [32] high contrast stationary stimuli produced greater responses than motion stimuli in V1, while the reverse was true in MT+. In summary, our data show that activations in primary visual regions evoked by contralateral motion stimuli are suppressed if bilateral central flow fields are presented. It seems that inhibitory inputs to primary visual regions cancel simultaneously occurring excitatory inputs. Since forward motion regularly induces bilateral optic flow stimuli continuous motion perception would be rather distracting. The observed non-activation in primary visual regions might hence reflect a functional decrement in the sensitivity needed to perceive motion [33]. Furthermore, our results indirectly support the assumption of a second pathway leading from the retina to MT+ perhaps via the SC [34], or a direct LGN input to MT+ [35], [36] without significant involvement of V1.

We localized MT+ on the ascending limb of the inferior temporal sulcus (corresponding to the junction of Brodmann areas 19, 37, 39). Comparing optic flow and static visual stimulation in the two visual hemifields we identified two subregions within the human MT+ complex on group level. One subarea was located more in the anterior part of MT+, being activated by ipsilateral and contralateral stimulation. This subarea supposably corresponds to MST (pMST). A second subarea was located in the more posterior part of MT+ (the non-MST part), and was activated exclusively by the contralateral stimulation. This subarea presumably represents MT. The location of subregions MT and MST from our group level data are in line with previous single subject data [16], [17], [37].

In addition, we found a hemispheric asymmetry in the MT+ complex in accordance with the findings of Brandt et al. [5] who mentioned a tendency for right hemispheric dominance in MT. In our data ipsilateral activation due to stimulation of the right visual hemifield was stronger than ipsilateral activation due to stimulation of the left visual hemifield. These results need to be confirmed.

While others reported great interindividual differences concerning the subregions of MT+ [37], our RFX group analysis largely compensated for interindividual differences and showed a robust delineation of MT and MST. Although data in a group level analysis require intensive processing and transformations, the random effects analysis as used in our data is supposed to allow for a generalization to the population level [38]. We did not only perform a region of interest analysis of the human MT+, but analyzed the whole brain. We could show that MT+ was the only region being activated by stimulation in the ipsilateral visual hemifield. In monkeys, receptive fields of MSTd neurons extend far into the ipsilateral visual field (up to 40°), while the receptive fields of MT cells only extend a few degrees (up to 10°–15°) into the ipsilateral visual field [39]–[42]. To avoid intermingling between MT and MST activations within the MT+ complex, some authors chose to set the edge of the ipsilateral stimulation pattern beyond the estimated distance, with which contralateral MT receptive fields might reach into the ipsilateral hemifield (about 10°–15° transferred from monkey studies; [16], [17]). In our approach, we placed the unilateral flow stimulus near to the centre (offset of edge of stimulation pattern 2°), i.e. well within the hypothesized receptive field size of contralateral MT cells. Surprisingly, we still measured two distinct and rather circumscribed subregions within the MT+ complex, one in the anterior part with ipsilateral stimulation, one in the posterior part with contralateral stimulation (the non-MST part). Of course, we cannot completely rule out that our anterior activation represents a mixture of MT plus MST activations. However, the locations of our subregions resemble those of the previous studies. We therefore tend to infer that activations of contralateral MT cells, whose receptive fields reach into the ipsilateral hemifield, play a minor role in this context. Furthermore, to our knowledge, there are no data available about receptive field sizes of MT cells in humans. The unscreened assumption that MT receptive field sizes can be transferred from monkey to human might therefore require reevaluation. Note that we did not find any contralateral activation outside the MT+ complex resulting from ipsilateral stimulation.

In conclusion, the primary visual cortex was not activated by central flow fields, but only by motion in the contralateral field. It appears that simultaneous stimulation of both hemifields by central flow leads to suppression of primary visual cortex activations. It remains to be shown if this mechanism is related to a functional reduction of motion perception. Moreover, we showed for the first time on group level that within MT+ a subregion can be identified corresponding to MST in the more anterior part of MT+. With such a group level approach it is possible to compare coordinates of related group results in MNI space. The comparison with previous studies showed that the eccentricity of the flow field relative to the mid-line plays a minor role for the location of the MT+ subregions. This result scrutinizes the assumed size of MT receptive fields in humans.
